# MR-Based PET Motion Correction Procedure for Simultaneous MR-PET Neuroimaging of Human Brain

**DOI:** 10.1371/journal.pone.0048149

**Published:** 2012-11-12

**Authors:** Marcus Görge Ullisch, Jürgen Johann Scheins, Christoph Weirich, Elena Rota Kops, Abdullah Celik, Lutz Tellmann, Tony Stöcker, Hans Herzog, Nadim Jon Shah

**Affiliations:** 1 Forschungszentrum Jülich, Institute of Neuroscience and Medicine 4, Medical Imaging Physics, Forschungszentrum Jülich GmbH, Jülich, Germany; 2 RWTH Aachen University, Department of Neurology, Aachen, Germany; 3 JARA Brain – Translational Brain Medicine, Aachen/Jülich, Germany; The University of Chicago, United States of America

## Abstract

Positron Emission Tomography (PET) images are prone to motion artefacts due to the long acquisition time of PET measurements. Recently, simultaneous magnetic resonance imaging (MRI) and PET have become available in the first generation of Hybrid MR-PET scanners. In this work, the elimination of artefacts due to head motion in PET neuroimages is achieved by a new approach utilising MR-based motion tracking in combination with PET list mode data motion correction for simultaneous MR-PET acquisitions. The method comprises accurate MR-based motion measurements, an intra-frame motion minimising and reconstruction time reducing temporal framing algorithm, and a list mode based PET reconstruction which utilises the Ordinary Poisson Algorithm and avoids axial and transaxial compression. Compared to images uncorrected for motion, an increased image quality is shown in phantom as well as in vivo images. In vivo motion corrected images show an evident increase of contrast at the basal ganglia and a good visibility of uptake in tiny structures such as superior colliculi.

## Introduction

Functional MRI (fMRI) is a common tool to assess brain networks by measuring the blood oxygen level dependent (BOLD) effect [1], which is a proxy for cerebrovascular changes caused by neuronal activation [2]. In the past, concomitant changes of the neuroreceptor/transmitter system had to be traced by PET in completely independent studies. Recently, the first hybrid MR-PET scanners have been introduced which allow simultaneous studies of magnetic resonance imaging (MRI) and positron emission tomography (PET) in humans [3], [4], [5]. The integration of MRI and PET in one instrumentation opens the way for novel multi-parametric studies where different aspects of brain function are observed by MRI as well as PET [6], [7]. Such studies are expected to have long acquisition times of more than one hour. Even when using appropriate motion-restrictions, head motion cannot be avoided during such long studies, as has commonly been experienced in both fMRI and PET. In neuroreceptor PET optical systems have been employed to track head motion and different approaches have been applied to correct for this motion [8], [9], [10], [11].

Alternatively, functional MRI image analysis software applications offer the possibility to track head motion by realigning the series of whole-brain image volumes acquired with an echo planar imaging (EPI) sequence at a temporal resolution of approximately 2–3 seconds. In combined fMRI/PET studies using simultaneous MR-PET, the motion parameters extracted from EPI may be exploited for correcting not only the fMRI results, but also the PET data [12].

Motion correction (MC) in PET brain studies may be based on the well-established multiple acquisition frame method (MAF) [13], [14], [15]. For each position of the subject the separately framed list mode data are reconstructed in the frame of reference given by the scanner. Consequently, this method needs only minor modifications of the workflow compared to the available standard reconstruction. Only the attenuation of the subject has to be modified by calculating motion adapted attenuation correction factors (ACFs) for each position. Since no transmission scan of the subject is possible for the BrainPET scanner, ACFs are derived from a template-based approach [16] using a co-registered subject MR image obtained by an MP-RAGE sequence. Additional attenuation due to the MR head coil in the field of view of the PET scanner is corrected using an attenuation image of the coil obtained from a Siemens ECAT EXACT HR+ PET scanner using a ^68^Ge transmission source. All other corrections applied to the data, e.g. normalisation and randoms correction, are applied in the same way as for the standard reconstruction. Finally, all reconstructed images are registered according to the known motion transformations. For the MAF method, the framing pattern of dynamic data is adjusted to the head movements. A drawback of this method is that multiple short frames must be separately reconstructed in the case of fast or frequent movement to minimise intra-frame motion.

To avoid this limitation, methods to perform MC at the level of line-of-responses (LORs) have been suggested [8], [17]. Nevertheless, the motion corrected LORs are usually filled and stored into classical sinograms to realise an efficient reconstruction as described by Catana *et al.* [12]. Here, the reduction of sinogram data in terms of span (axial compression) and mashing (transaxial compression) as well as the use of the concepts of interleaving and direct/indirect planes compromise the data prior to reconstruction. This leads to unavoidable image degradation. In order to utilize LOR-based motion correction (LORMC) in a better way the fully 3D set of all LORs must be taken into account without significant data compression.

In this context, the PET Reconstruction Software Toolkit (PRESTO) is a new software framework for fully 3D iterative PET image reconstruction using scanner-independent, adaptive projection data and highly rotation-symmetric voxel assemblies [18]. A memory-resident, pre-calculated system matrix based on Volume-of-Intersection calculations [19] is realised due to the exploitation of rotational symmetries for efficient matrix compression. PRESTO utilises a generic ring detector onto which the actual PET scanner is mapped. This feature offers a way to include head motion data at the line-of-response level in the image reconstruction without using ‘classical’ sinograms and data compression. By this, the true sampling pattern of the scanner is taken into account more accurately. Also the normalization of corrected LORs and the out-of-field problem of LORs leaving or entering the field of view (FOV) after motion correction can be consistently handled. Compared to a classical sinogram-based reconstruction as used by Catana *et al.* [12], PRESTO provides an improved resolution-noise trade-off at cost of higher computational burden [18]. Therefore, an efficient framing algorithm, which minimises the trade-off between intra-frame motion and total number of motion frames becomes of great importance. This is especially true for the MAF method which requires separate reconstruction of any such motion frame.

A new framing algorithm based on two assumptions was developed. Firstly, when utilising a frame-based motion reconstruction, residual motion within the frame reduces the image quality. Thus, the problem of finding optimal framing involves the task of minimising residual intra-frame motion. Secondly, head motion can be coarsely divided into two types a) rapid head movements that occur at position changes within short time frames, and b) slow drifting motions, as well as there being a continuous transition between the two types of motion. An example of motion data showing this can be found in Figure 3.10 of [11]. An example of a fast motion would be head motion triggered by other motor activity such as response inputs during an fMRI study, or the rapid head movements occurring in Tourette patients during a tick. An example of slow motion is the drift that can often be observed due to neck muscles relaxing over the course of the examination. In this case, depending on the patient bed, the head slowly tilts towards or tips away from the chest as the muscles relax.

A well-known drawback of EPI is image distortion caused by the long echo train readout. Parallel imaging techniques such as GRAPPA [20] can shorten the readout and reduce image distortion. Thus, we investigated the effect of parallel imaging on motion estimation accuracy and provide an EPI protocol optimised for high accuracy motion parameters also suitable for most fMRI experiments.

The present paper combines optimised MR-based motion tracking, an error minimising, object specific temporal framing algorithm, and a list mode based reconstruction within the PRESTO environment to consider motion data during the iterative reconstruction of fully 3D LOR data. The method is applied to ^18^F phantom data and *in vivo* ^18^F-FDG data and compared to the multiple acquisition frames (MAF) reconstruction. In this way we examined whether an increased image quality can be obtained by the motion correction method presented here.

## Materials and Methods

### System Description and Basic Methodology

All measurements were performed on a hybrid MR-BrainPET scanner consisting of a Siemens 3 Tesla Tim-Trio system with an integrated BrainPET insert (Siemens Healthcare, Erlangen, Germany). MR acquisition was performed with a dual coil, produced by Siemens Healthcare, consisting of two parts: a single channel birdcage element used for both excitation and reception and an eight channel phased array, fitted inside the birdcage coil used for signal reception only. The BrainPET insert consists of 32 detector cassettes organised in a ring with an inner diameter of 37.6 cm and an axial FOV of 19.3 cm. In each cassette 6 detector blocks with a 12×12 Lutetium Oxyorthosilicate (LSO) crystal array (2.5×2.5×20 mm^3^ crystal size) are read out by 9 avalanche photo diodes. The BrainPET offers an optimal central resolution of approximately 3 mm [4]. All PET data were recorded in list mode. Synchronisation between MR and PET was achieved by feeding the output trigger of the MR scanner into the PET trigger signal port. The broadcast trigger signal indicating the beginning of the next EPI volume acquisition instantaneously causes a trigger tag word to be written into the chronological stream of list mode events with an accuracy of 0.2 milliseconds [4]. Motion correction was achieved with a new method consisting of four steps, described in detail in the following sections: (1) calibration of the system position offsets between MR-scanner and PET system, (2) motion parameter extraction from MR images, (3) subdivision of PET data into discrete parts referred to as frames, and (4) motion correction of list mode data with subsequent image reconstruction.

### Registration of Coordinate Systems

In order to apply the motion parameter information extracted from MR images in the PET reconstruction process, the relative position offset between the iso-centre of the MR scanner and the BrainPET insert has to be measured. This calibration was performed by means of mutual information co-registration [21] of a simultaneously acquired MP-RAGE (1×1×1 mm^3^ voxel size, detailed parameters in Table 1: MP-RAGE) and a ^18^F PET image (1.25×1.25×1.25 mm^3^ voxel size in a 256×256×153 matrix) of a two-chamber phantom [22]. The phantom is a straight cylinder of 10 cm length and a head shaped outline with two chambers roughly mimicking grey and white matter outlines of the brain. This measurement was performed for six unique phantom positions and the resulting offsets were averaged to minimise uncertainties. However, the calibration step is not limited to the method described here. In principle, methods such as the one described by Langner in [11] can also be utilised.

**Table 1:** Sequence protocols.

	MP-RAGE	EPI1	EPI2	EPI3	EPI4
**Matrix Size**	256×256×176	64×64×45	64×64×45	64×64×45	64×64×45
**Voxel Size [mm]**	1.0×1.0×1.0	3.7×3.7×3.7	3.7×3.7×3.7	3.7×3.7×3.7	3.7×3.7×3.7
**Flip Angle [°]**	9	83	82	81	80
**TE [ms]**	3.03	30	30	30	30
**TR [ms]**	2250	2730	2400	2290	2220
**TI [ms]**	9	-	-	-	-
**GRAPPA Factor**	2	no PI	2	3	4

Sequence protocols.

### Motion Extraction and Frame Definition

Motion information, represented as 6 parameters describing rigid body motion with 3 translations and 3 rotations, was extracted from EPI [23] time series data using the Statistical Parametric Mapping (SPM) realignment algorithm [24]. The motion data were separated into individual frames using a framing algorithm which minimises the residual intra-frame motion.

**Figure 1:**
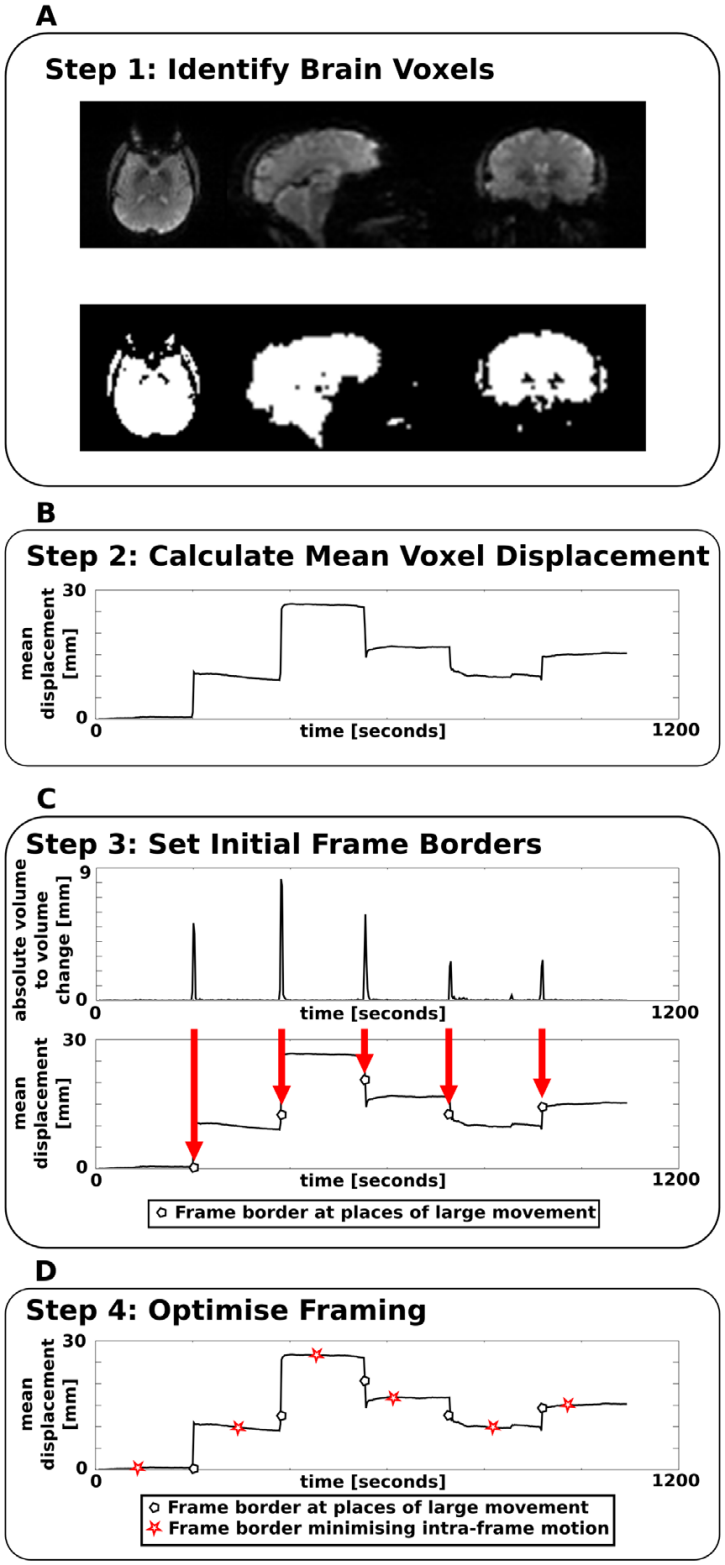
Framing algorithm schematic. A schematic overview of the proposed framing algorithm which consists of four main steps. Step 1: identification of brain voxels from the first acquired EPI volume. (A) The top row shows EPI images and the bottom row shows the resulting binary mask with brain voxels marked in white. Step 2: calculation of the mean voxel displacement of the brain for each time-point of the EPI time series. (B) An example mean voxel displacement trace is shown. Step 3: set initial frame borders where a large change in displacement occurred between two consecutive scans. (C) The top graph shows the absolute volume-to-volume change of the mean voxel displacement. The bottom row shows the resulting frame borders overlaid on the mean voxel displacement. Step 4: Minimise intra-frame motion by setting of additional frame borders such that the intra-frame motion is minimal. (D) The resulting frame borders are shown as red stars overlaid on the mean voxel displacement trace.

The framing algorithm performs the four steps which are schematically presented in Figure 1. A detailed description of each step is given below.

Step 1 (Figure 1A): identification of all brain voxels is performed by k-means clustering [25]. Intensity values from all voxels in the first EPI volume are separated into two clusters, one containing background pixels and one containing the brain. The use of k-means clustering for brain extraction is advantageous for minimising operator interaction. Step 2 (Figure 1B): for each brain voxel, *i*, in volume *n* of the EPI time series, a scalar displacement distance 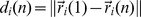 in millimetres as proposed in [11] is calculated relative to the voxels initial position 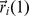 in the first scan (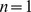) of the EPI time series (*n = 1..N*). A mean voxel displacement of the brain, 
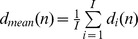
, relative to the voxel position in the first scan is calculated for each time-point *n*. Step 3 (Figure 1C): the absolute volume-to-volume change between two consecutive time-points 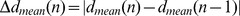 is calculated. Local maxima (peaks) in the displacement between two consecutive time points are detected. Frame borders are set at the positions of peaks if the movement was larger than a predefined threshold (1 mm) and separated by at least a predefined minimum frame length (1 min) from the nearest frame border. These thresholds were empirically chosen; 1 mm motion as this is one third of the PET system resolution at the centre of the field of view [4], and a minimum frame length of 1 minute as a trade-off between computation time and motion artefact reduction. Step 4 (Figure 1D): this coarse framing is then refined in a second framing step: each frame longer than twice the specified minimum frame length is subdivided at the time-point minimising the mean voxel displacements *within* the sub frames relative to the mean frame position. The mean frame position is calculated by individually averaging the six motion parameters (translations along the *x*, *y*, and *z* axes (mm), and the three Euler rotation angles α for rotations around the *x*-axis, *β* for rotations around the *y*-axis, and γ for rotations around the z-axis (degree)). While this averaging is not entirely mathematical correct due to the combinatorial relationship between the parameters, comparisons to voxel-wise Euclidian coordinate averaging showed reduced computation time without affecting the results of the framing.

The coarse, first framing step of the algorithm sets frame borders for the case where the patient's head moved rapidly into a different position and the second framing step reduces the influence of slow motions.

### PET Image Reconstruction without Motion Correction

For image reconstruction the Ordinary Poisson Algorithm (OPA) [27] was applied within PRESTO [18]. The OPA advantageously incorporates additive and multiplicative correction terms in the iteration update formula, thus preserving Poisson statistics and taking the non-negativity constraint appropriately into account [27]. Thus, data pre-corrections, e.g subtraction of the additive random/scatter background, which always produce a higher level of noise as well as a bias in the images, are omitted completely.

**Figure 2:**
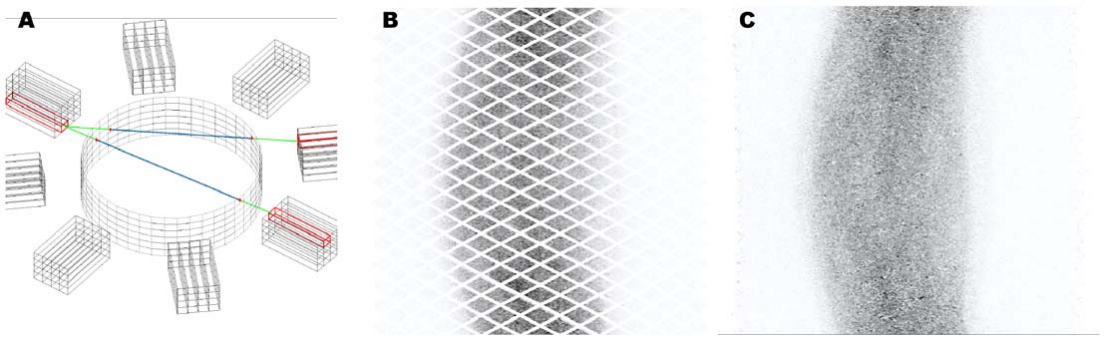
Generic projection data. (A) Sketch of the 3D data conversion between physical LORs and generic LORs in PRESTO, the physical detector blocks (outer part) define LORs that can be interpolated to the Generic Cylinder (inner part). Intersection points (red dots) pick up unique generic crystal combinations. (B) Generic projection data without considering subject motion. (C) Generic projection data with applied LORMC motion correction. For simple visualisation the projection data are sorted for view angle (vertical) and radial coordinate (horizontal) according to classical sinogram terminology.

The procedure of PRESTO to convert measured data into generic data is sketched in Figure 2A. Any physical Line-of-Response (LOR) is uniquely assigned to a specific generic crystal combination by calculating intersection points between the physical LOR and the cylinder surface. These two intersection points define a unique combination of generic crystals for which the iterative reconstruction holds an accurate projector [19] ready. Further details can be found in [18]. A computationally optimised version of the PRESTO software was used [28].

For the BrainPET detector providing approximately 280 million physical LORs (see system description above) the adjusted generic setup considers 480 million independent LORs within the reconstruction. This significant discrepancy is motivated by the fact, that the BrainPET scanner includes several detector gaps which are modelled as generic crystals as dummy placeholders (see Figure 2).

Detector sensitivities are derived from specific normalisation measurements, and individual variance-reduced normalisation factors *N*(*i,j*) are derived for all reasonable detector pairs (*i,j*) [29]. Data correlations as well as all existing spatial symmetries are used to apply a decomposition of *N*(*i,j*) into a pure geometrical component *G*(*i,j*) directly depending on the detector pair (*i,j*) and additional intrinsic crystal efficiencies *E*(*i*) resp. *E*(*j*). Thus, normalisation factors *N(i,j)* can be empirically factorised into individual variance-reduced components as follows:
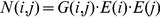
(1)Using the factorisation of Eq. (1), numerical values of all components are determined from the measured plane source data by minimising the mean square differences between measured and predicted values.

Random events *R^meas^(i,j)* are directly measured for each detector pair using the delayed window technique. In addition, Variance Reduction (VR) of the random rates is applied in a post-processing step [30], i.e. *R* ^meas^(*i,j*)⇒*R^VR^*(*i,j*). The three independent components of any physical detector pair, i.e. detected prompt events *P(i,j)*, variance-reduced random events *R^VR^(i,j)*, and normalisation *N(i,j)*, are separately assigned to the generic projection space by addressing generic crystal pairs *(k,l)*. The applied mapping (*i,j*)⇔(*k,l*) between physical and generic crystal pairs depicts an unambiguously reversible function Ψ(*i,j*) = (*k,l*) with the immediate identities *N*′(*k,l*) = *N*(*i,j*), *P*′(*k,l*) = *P*(*i,j*) and *R*′(*k,l* ) = *R^VR^*(*i,j*) for corresponding pairs.

The attenuation images (μ-maps) of the subject and the MR coil undergo a forward projection into the generic projection space to obtain relevant ACFs *A^head^(k,l)* and *A^coil^(k,l)*. Thus, the effective sensitivity is given by
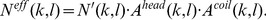
(2)


### PET Motion Correction

The implemented PET motion correction is founded on the motion extraction and optimised framing definition as described in the previous section. For any required motion frame ε = {1, …, *N*_frame_} the transformation matrix ***M***_ε_, a matrix representation of the measured motion data, allows the compensation of motion. Using ***M***_ε_ two different approaches for the motion correction, MAF and LORMC, of the acquired list mode data are realised and compared.

### Multiple Acquisition Frame (MAF) Method

Using MAF [13], [14], all frames can be independently reconstructed as described above. Only the subject's ACFs *A* ^head^(*k,l*) have to be adapted for every frame. Matching values *A* ^head^(*k,l,*ε) are calculated separately for every frame ε by transforming the subject's initial attenuation map according to ***M***_ε_ previous to forward projection. Finally, the reconstructed image for each frame ε is transformed using (***M***_ε_)**^−1^** to provide a set of correctly registered images, which can be superposed.

### LOR-based Motion Correction (LORMC) Method

In LORMC, motion of the subject with respect to the scanner can be compensated by applying an inverse transformation of the whole scanner before filling the generic projection space for each frame separately. The 3D coordinates of all physical detectors are transformed from the nominal position **x**_d_ to the modified position **x**_d_′(ε) for any frame ε according to
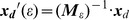
(3)with 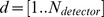.

Then, instead of the nominal coordinates the modified coordinates **x**_d_′(ε) are applied to fill the generic projection space with the acquired data of the physical detector combinations (*i,j*). This means, for each frame ε a specific physical detector combination (*i,j,*ε) is assigned to a specific generic combination (*k,l*) depending on the corresponding transformation ***M***_ε_ respectively, i.e. Ψ(*i,j,*ε) = (*k,l*). This is similar to the approach in [12], but now a generic projection space is filled instead of sinograms. Prompt events and variance-reduced randoms can be separately integrated in the generic projection space as follows:
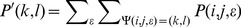
(4)

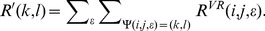
(5)


Using the motion-corrected integrated prompts and randoms Eq. (4) and (5) require appropriate normalisation factors which can be calculated according to the post-normalisation procedure as described in [31]. Note, effective post-normalisation factors *N* ^post^(*k,l*) are given by integrating sensitivities [26] rather than averaging normalisation factors of contributing LORs. The frame-invariant detector sensitivity is defined as
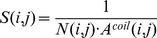
(6)and the total sensitivity of a generic LOR is calculated as follows:
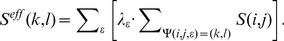
(7)


The value λ_ε_ gives the statistical weight of each frame according to the frame duration and appropriate decay correction factor. In fact, count rate reduction during the measurement related to the half-life time of the tracer isotope effectively means a loss of global sensitivity. Hence, statistical weights of different frames have to take decay correction factors into account to obtain unbiased time-integrated sensitivities, which are conform to Poisson statistics.

Attenuation of the subject has only to be considered in the frame of reference because of the applied motion correction of all LORs. Finally, in contrast to Eq. (2) post-normalisation factors are now given as
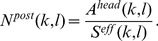
(8)


### PET Data Processing

PET data processing and image reconstruction was performed on a multi-user cluster equipped with 5×2 Intel Xeon X5365 3 GHz CPU (double Quad Core architecture) with 24 GB RAM per board. Forward projections as well as complete iterative reconstruction of PET images with PRESTO using the OPA were performed using 6 threads in parallel. In total, all 480 million generic LORs are considered corresponding to a size of the compressed system matrix of 17 GB.

For the MAF method, attenuation caused by the subject requires the forward projection of an adapted, motion-corrected μ-map and subsequent calculation of ACFs for each frame. In contrast, for the LORMC method only a single calculation of ACFs is required in the frame of reference. However, the LORMC mapping function 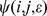 has to be calculated for each position to map each of the 230 million physical LORs to the generic projection space. Once the mapping function is calculated, the corresponding list mode data can be sorted (1 thread) separately for the prompts, randoms, and sensitivity. Every filling step requires the same number of operations for MAF and LORMC.

### PET Image Quality Assessment

**Figure 3:**
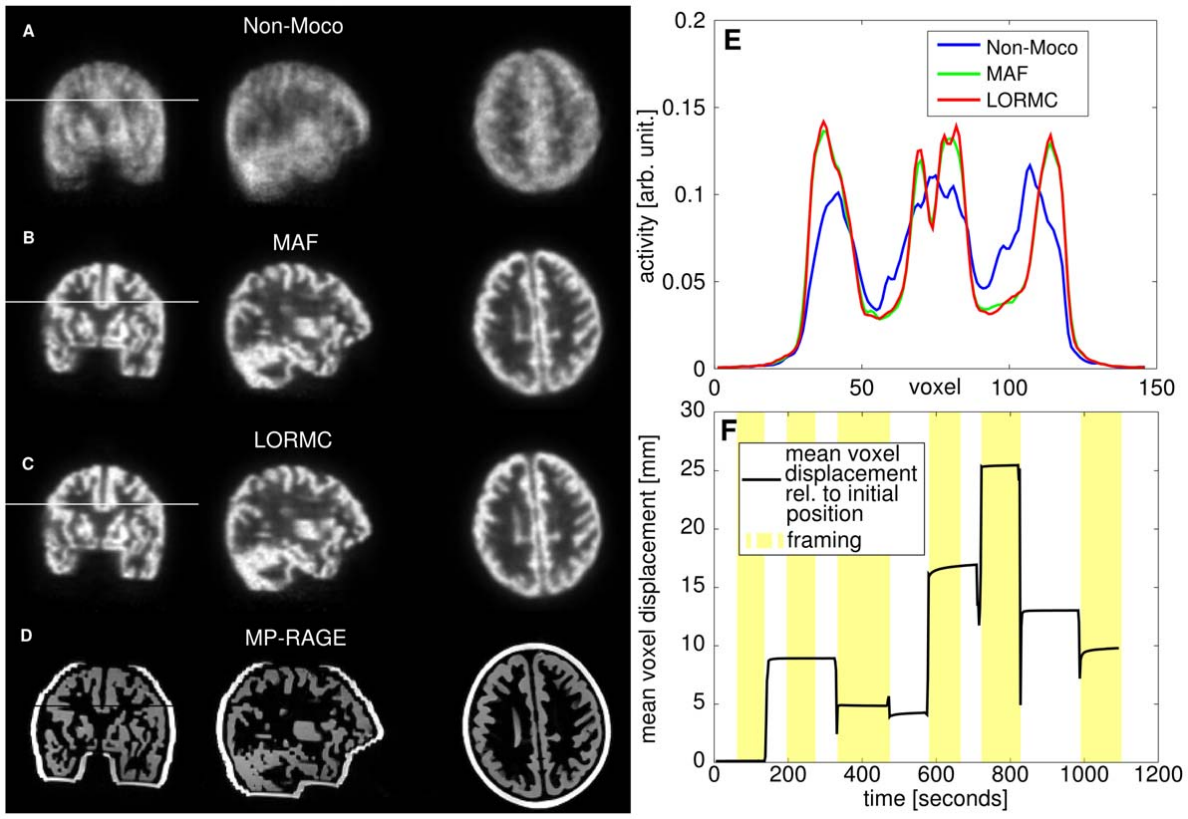
Iida Brain Phantom. (A) Non-motion corrected, (B) MAF corrected, (C) LORMC corrected PET images, and (D) MP-RAGE image of the Iida brain phantom filled with ^18^F doped water. (E) On the right the profiles along the white lines in the images (A–C) are plotted. (F) Shows the patient motion quantified as the mean voxel displacement relative to the initial position and the 12 frames used in the motion corrected reconstructions. The blurring due to motion is reduced in the motion corrected images.

The PET image quality obtained with either MAF or LORMC method was compared by evaluating the sum of mean square differences (MSD) with respect to an accurate, matching reference image under the terms of
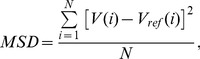
(9)with *V(i)* and *V_ref_(i)* defining the corresponding voxel values of both images with a total of *N* voxels. For acquired patient or phantom data the true distribution is not exactly known. However, when comparing different reconstruction methods using samples with very low number of counts, a sample with a high number of counts can be reconstructed and regarded as valid reference image. In this way, remaining uncertainties from the high statistic image are negligible with respect to the significantly higher fluctuations to be quantified. Here, we are especially interested in the quantification of possible (relative) deviations between images of the MAF and LORMC method in case of short single position frames (<4 seconds) of real data. Therefore, from the Iida phantom measurement with 12 distinct localisations during a total acquisition time of about 20 minutes, artificial frames of 3 seconds have been extracted for each position from list mode data. Combining 12 such frames, i.e. a single 3 second frame of each phantom position, effective images have been reconstructed using the LORMC method and MAF method respectively. As a reference image, the motion corrected reconstructed image of the complete data is used (Figure 3C). Then, the MSD values can be evaluated as a function of the number of iterations. Additionally, the reproducibility of the results is verified by artificially generating further statistical independent frames for each position with subsequent analysis in same manner.

### Measurements

#### Coordinate Calibration Measurement

All coordinate calibration measurements were performed using a two-chamber phantom [22], consisting of two chambers with volumes of 450 ml for the inner chamber and 600 ml for the outer chamber. The phantom was filled with distilled water containing a total of 40–80 MBq of either ^18^F or ^18^F-FDG and 0.2 ml of Gadopentetate Dimeglumine. The concentration ratio between the inner and outer chamber was approximately 1∶4. The phantom was imaged in six unique positions with an acquisition time of 10–15 minutes per position.

### Motion Estimation Accuracy

To assess the accuracy of the motion estimation achieved with the EPI sequence, a preliminary study was performed with a polymer phantom, referred to as the Iida Brain Phantom [32]. This phantom mimics the human brain by offering two compartments, modelling the grey matter and skull, respectively, of a young healthy volunteer. It is constructed from a photo-curable polymer with density of 1.07 g/ml by using a laser-modelling technique. Although the space inside the grey matter compartment is called “white matter” below, it consists of polymer and cannot be filled with radioactivity.

It is well known that the use of parallel imaging in MRI can reduce image distortions in EPI. To investigate whether the use of parallel imaging affects motion measurement accuracy, we measured with different EPI protocols utilising varying degrees of parallel imaging by changing the GRAPPA [20] acceleration factor. The phantom was placed in the scanner in 10 different positions. In each phantom position, an MP-RAGE image with 1×1×1 mm^3^ voxel size and four EPI protocols with varying GRAPPA [20] factors R = (1, 2, 3, 4) were acquired as separate acquisitions. A complete list of acquisition parameters is given in Table 1. At each phantom position ten EPI volumes were acquired for each EPI protocol.

Reference motion parameters were generated by realigning the MR-RAGE images of each phantom position using SPM [24]. For each EPI protocol ten sets of motion parameter data were extracted by realigning the n-th (n = [1..10]) EPI volume acquired in each position, 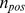. Using the extracted motion parameters the coordinates of the image voxels containing the phantom were calculated for positions two to nine for the MP-RAGE reference and the four EPI protocols. For each voxel, 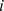, a scalar voxel position error 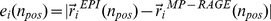 was defined, and the mean voxel position error over all voxels was calculated. Finally, the root-mean-square (RMS) of the mean voxel position error over the nine positions was calculated.

### MR-PET Phantom Study

Using the EPI protocol with the optimal GRAPPA factor, a phantom study was performed using the Iida phantom. The grey matter compartment was filled with a solution containing approximately 140 MBq of ^18^F and 0.2 ml of Gadopentetate Dimeglumine. The phantom was positioned in the iso-centre of the PET scanner and MR and PET data were acquired simultaneously. An MP-RAGE with 1×1×1 mm^3^ voxel size (see Table 1: MP-RAGE) was acquired with the phantom in its reference position. Following the MP-RAGE scan, an EPI time-series was measured continuously for 18 minutes (see Table 1: EPI4) in a single acquisition. During the acquisition of the EPI images, the phantom was moved to different positions at irregular intervals of 2–5 minutes over the course of the entire scan. An attenuation map of the phantom was measured on an ECAT EXACT HR+ (Siemens/CTI, Knoxville, TN, USA) PET scanner with a transmission scan time of 10 hours using a ^68^Ge source. Three sets of PET images were reconstructed: 1) uncorrected for motion, 2) one set with MAF motion correction and 3) one set corrected for motion with the LORMC method.

### MR-PET In vivo Study

*In vivo* measurements were performed in three patients without brain disease. The patients were injected with 310 MBq (Patient A), 320 MBq (Patient B) and 330 MBq (Patient C) of [^18^F]-fluoro-deoxy-glucose (FDG) and received a whole-body PET examination for oncological diagnostics in a conventional PET system (ECAT EXACT HR+). After the whole-body measurement and starting between two to three hours after injection the patients were measured in the hybrid MR-PET scanner without receiving additional radioactivity. An anatomical scan was performed with the MP-RAGE protocol described in Table 1. Thereafter, a functional scan using the EPI sequence (Table 1: EPI4) with 500 volumes and a total acquisition time of 18 minutes was acquired. Every 1–3 minutes the patients were instructed to move their heads into a different position. Attenuation maps were created using the method described in [16] and three sets of images were reconstructed from the data: 1) standard PET reconstruction without motion correction, 2) reconstruction with the MAF motion correction method, 3) reconstruction with the LORMC method described above. A reference PET image, not subject to induced motion, was reconstructed from the data acquired during the measurements of the anatomical MR sequences. During this time of approximately 15 minutes the patients were instructed not to move.

Written informed consent was obtained prior to measurement. The study was approved by the ethical committee of the Heinrich Heine University at Düsseldorf, Germany, and was conducted under the conditions of a clinical study according to §20 of the German Medizinproduktegesetz (German Act on Medical Devices).

## Results

### Coordinate Calibration

The standard deviation of the coordinate calibration results was measured to be 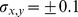 mm for translations along *x* and *y*, 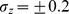 mm for translations along *z*, 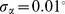 for rotations around the *x*-axis, and 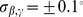 for rotations around the *y*- and *z*-axes.

### Motion Estimation Accuracy

**Figure 4:**
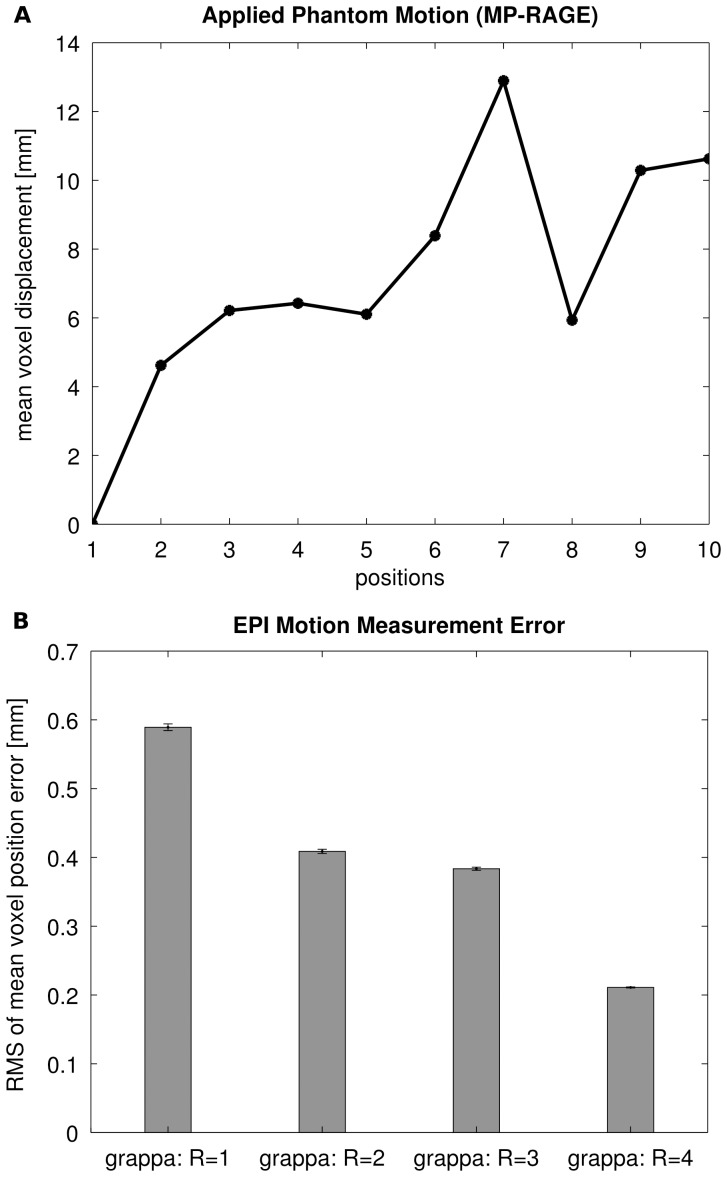
Motion estimation accuracy. Phantom study on the influence of parallel imaging on the accuracy of the motion parameters. (A) Mean voxel displacement relative to the initial position calculated from the motion parameters extracted from the MP-RAGE measurements of the Iida Brain phantom. (B) RMS of the mean voxel position error for the four EPI protocols measured with GRAPPA factors of R = 1 to R = 4. The mean of ten measurements is shown. Error bars mark the standard deviation. Please note the error bars being small due to a maximum relative standard deviation of 0.8%. Using GRAPPA increases the accuracy of the measured motion parameters.

Figure 4A shows the mean voxel displacement calculated from the reference motion parameters acquired from the MP-RAGE measurements with the Iida brain phantom. Figures 4B depicts the RMS of the voxel position error for the four measured EPI protocols. The error bars represent the standard deviation of the root-mean-square error over the ten repetitions of the measurements.

The RMS of the voxel position error reduces from nearly 0.6 mm without the use of parallel imaging to 0.2 mm with a GRAPPA factor [20] of R = 4. Consequently, a GRAPPA factor of R = 4 was applied for measurements reported here.

### Framing Algorithm

**Figure 5:**
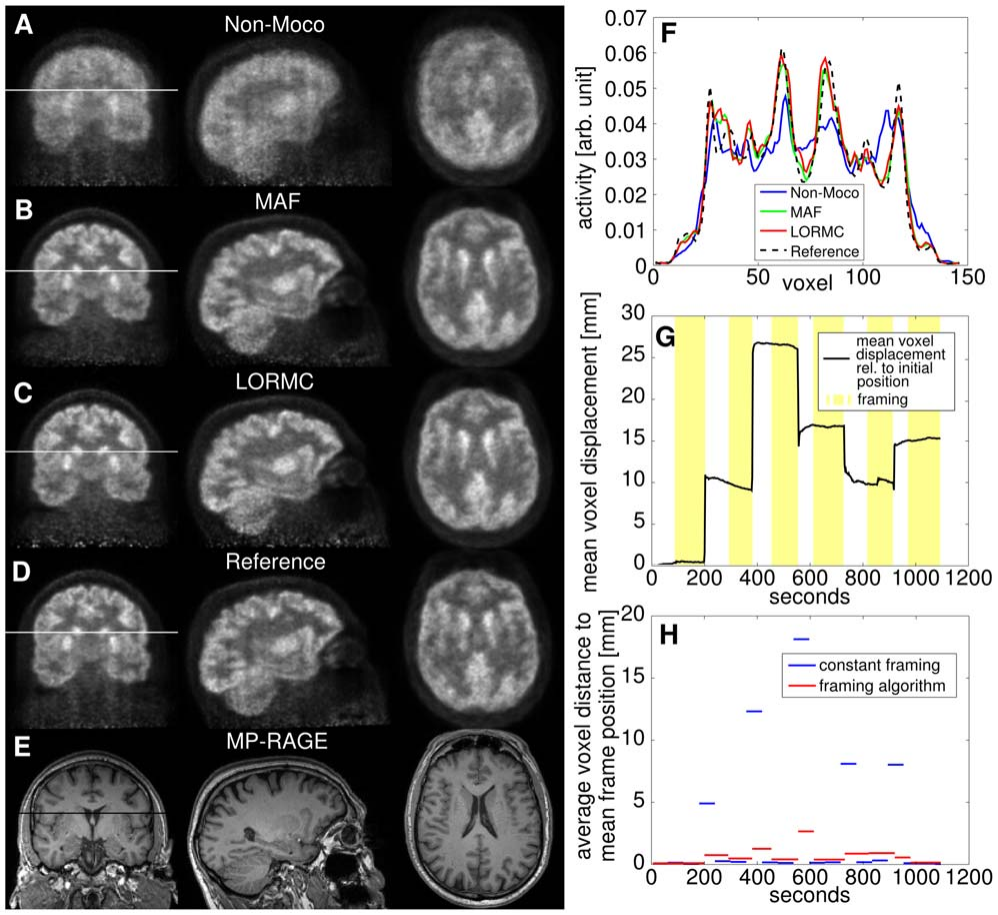
Patient A. (A) Non-motion corrected PET image, (B) MAF corrected PET image, (C) LORMC corrected PET image, (D) reference PET image acquired without induced motion, and (E) MP-RAGE image of Patient A. The profiles along the white lines in the images A–D are shown in (F). (G) Subject motion quantified by the mean voxel displacement relative to the initial position is displayed as a black line (see step 2 of the framing algorithm for details). Yellow and white horizontal stripes mark the framing calculated with the framing algorithm consisting of 12 frames. (H) Depicts the residual intra-frame motion for regular 1 minute framing and algorithmic framing. The residual intra-frame motion is quantified for each frame by the average voxel displacement relative to the mean frame position. For regular 1 minute framing (blue) the residual motion inside the frame is larger than for the automated framing algorithm (red). The width of the horizontal line denotes the frame length. The use of the framing algorithm reduces the average voxel distance to the mean frame position from.

As an example, the framing of Patient A and the residual mean voxel displacement in the frames, a measure for the residual intra-frame motion, are shown for two framing schemes in Figure 5H. Blue lines in Figure 5H denote the residual mean voxel displacement of a regular framing with a frame length of one minute. Red lines in Figure 5H show the residual mean voxel displacement of the frames calculated by the automated framing algorithm. The lengths of the lines denote the frame lengths. For all frames combined the residual mean voxel displacement with regular framing is 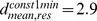 mm for 19 frames. The framing algorithm reduces this to 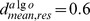 *mm*, well below the system resolution of 3 mm, while additionally reducing the number of frames to 12.

### PET Data Processing Performance

For the LORMC method the calculation of the mapping function 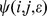 takes 37 seconds for each position (1 thread). Once, the mapping function is calculated, the data sorting (1 thread) needs approximately 8 seconds calculation time respectively for the prompts, randoms, and sensitivity, i.e. 24 seconds for completion of any frame. The calculation of ACF values needs approximately 30 seconds (6 threads), which is required only once for LORMC, but for any frame in case of MAF. Finally, iterative reconstruction takes approximately 53 seconds (6 threads, 2 subsets) per subset.

Figures 2B and 2C illustrate generic projection data without considering subject motion (B) and applying LOR-based motion correction according to the LORMC method (C). The acquired list mode data of the brain measurement in one of the three patients is converted to the generic projection space and normalised. For the LORMC method (C) 14 different positions and frames have been processed and filled. Due to the motion of the subject, the detector gaps visible in the case of a stationary filling (B) are successively filled by physical LORs. Note, for visualisation the generic projection data are sorted for view angle (vertical) and radial coordinate (horizontal) according to classical sinogram terminology.

### MR-PET Phantom Study

The induced motion of the phantom leads to a deteriorated image quality in the uncorrected image (Figure 3A), and a reduction in contrast between the grey and white matter. In the motion-corrected images depicted in Figure 3B (for MAF) and Figure 3C (for LORMC), no blurring is visible and the contrast between grey and white matter is higher compared to the non-motion corrected image.

**Figure 6:**
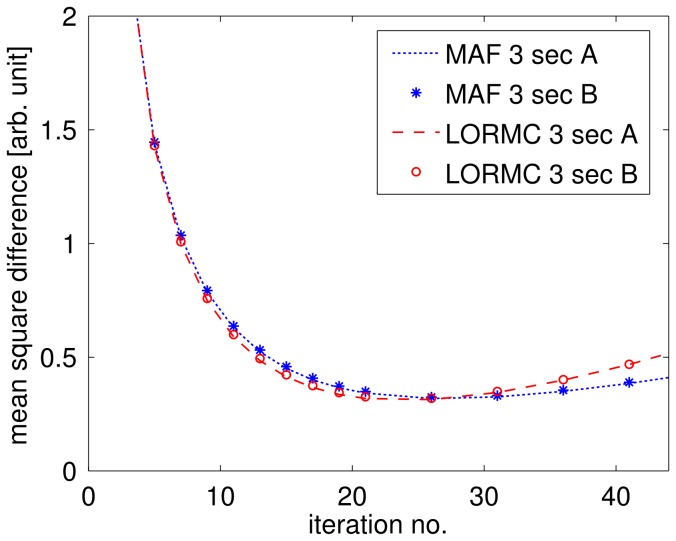
Mean square difference analysis. Mean Square Differences as function of the number of iterations for the Iida phantom measurements in 12 different positions reconstructed with the LORMC and MAF methods. As reference image with high statistics the image of Figure 4C is used for all evaluated low statistic images (3×12 s).

Figure 6 provides a quantitative comparison of the image quality in terms of MSD values between MAF and LORMC method in case of low count statistics using the Iida phantom data. MSD curves are plotted as function of the number of iterations for reconstructed images based on 12 positions of the phantom effectively considering 12×3 seconds of acquired data. Generally, all curves show a global minimum of the MSD value indicating the best achievable correspondence to the reference (Figure 3C). In agreement with a visual inspection of the images comparable results between MAF and LORMC method are observed using identical framing schemes. Additionally, for frames with low count rates MAF and LORMC provide an equivalent image quality. However, LORMC reduces the necessary computation time. Finally, additionally generated statistically independent samples (blue asterisk and red circle) provide nearly identical curves concluding that evaluated MSD values are highly reproducible and sensitive to possible differences in performance.

### MR-PET in vivo Study

**Figure 7:**
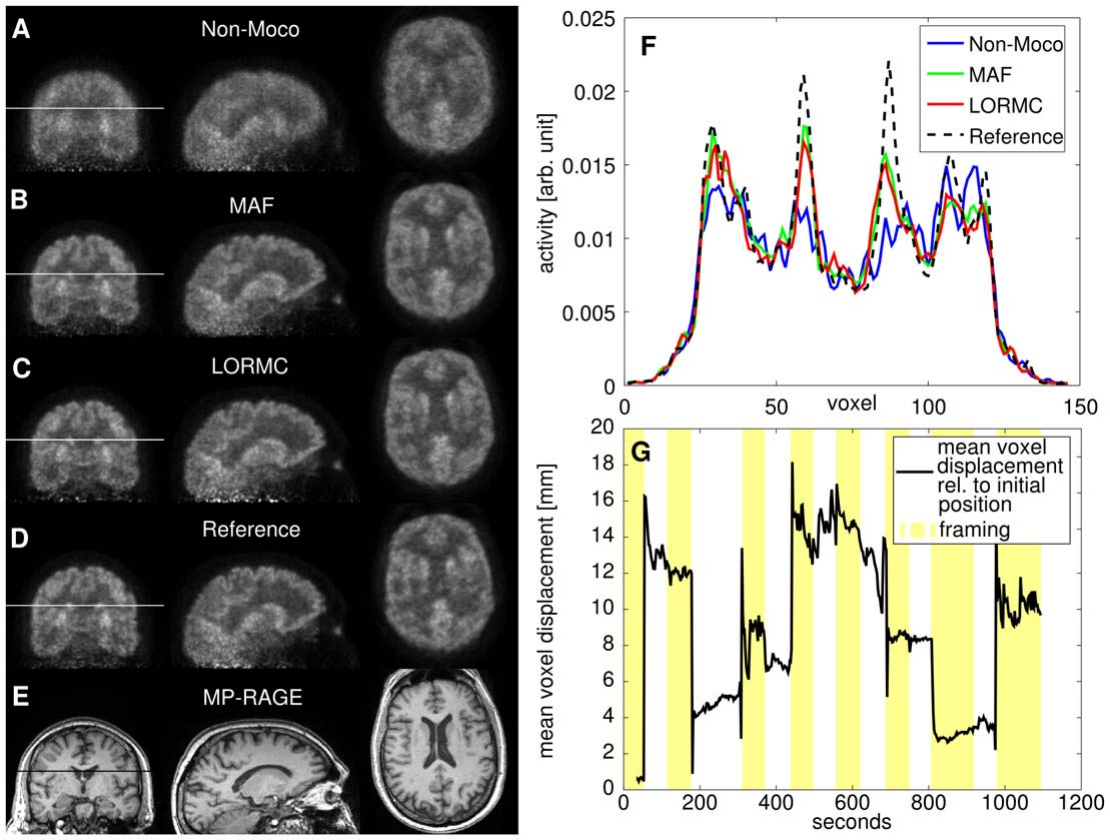
Patient B. (A) Non-motion corrected, (B) MAF corrected, and (C) LORMC corrected PET images, (D) reference PET image without induced motion, along with (E) the corresponding MP-RAGE image of Patient B. The profiles (F) along the white lines in the images A–D are shown in the top right corner. (G) Patient motion is shown as mean voxel displacement relative to the initial patient position - the metric calculated in the second step of the framing algorithm.

Figure 5 shows data from a patient where minimal motion occurred between induced motions, while the patient in Figure 7 exhibited substantial intra-frame motion. Data from a third patient with average intra-frame motion is shown in Supplemental Figure 1. The figures depict (A) non-motion corrected, (B) MAF corrected, (C) LORMC corrected, and (D) reference PET images without motion, along with (E) the corresponding MP-RAGE slices. (F) In the top right-hand corner, a profile along the white lines drawn in the images is shown for the non-motion corrected and the motion-corrected PET images. In (G) the calculated mean voxel displacement of the brain relative to its initial position (see also step two of the framing algorithm for details) is shown. The motion recorded by the EPI tracking differs slightly between the two patients: in one patient (Figure 5) there was only little motion between the instructed large movements, while the other patient (Figure 7) showed trembling movements and drift between the instructed large movements. Data from a third patient is depicted in Figure S1.

**Figure 8:**
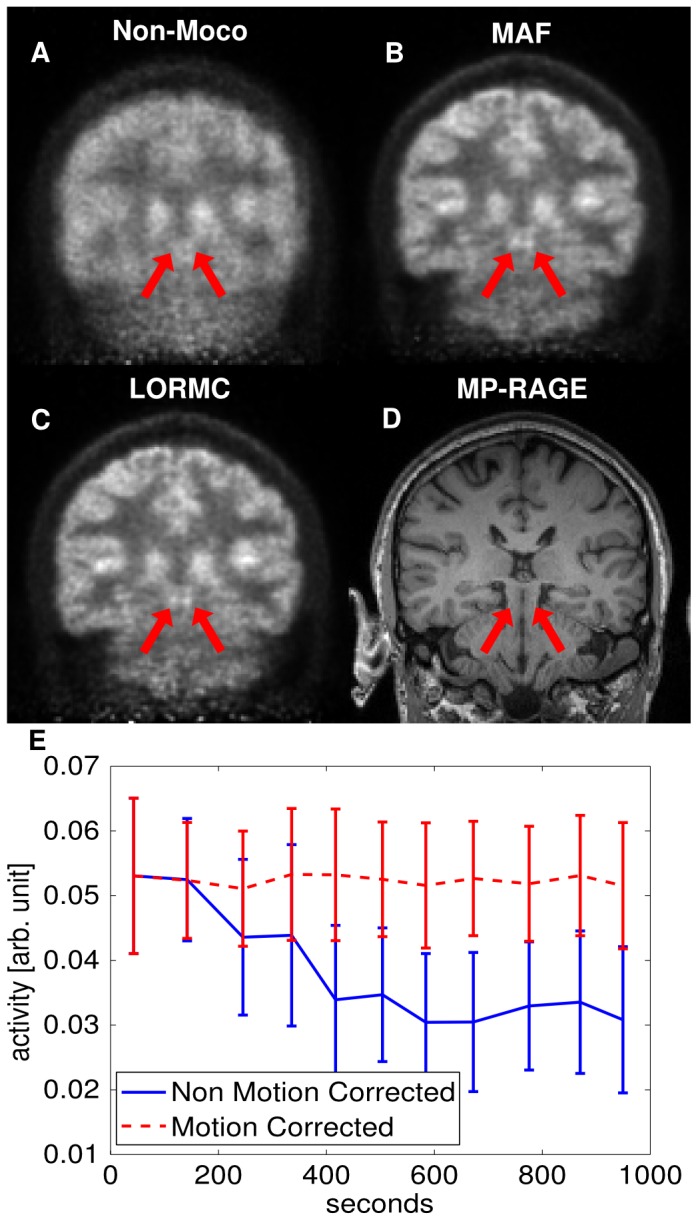
*Superior colliculi* and tracer dynamics of Patient A. (A) Non-motion corrected image. (B) MAF corrected, (C) LORMC corrected PET images of Patient A, and (D) the corresponding MP-RAGE slice. The red arrows mark the *superior colliculi*. Uptake is visible only in the motion corrected PET images. (E) Dynamic study of the uptake in the *basal ganglia*. Error bars show the standard deviation of uptake values in the region of interest. The motion corrected (MAF) time activity curve appears more stable than the non motion corrected time activity curve.

The non-motion corrected images show severe blurring artefacts due to the large motions occurred during the acquisition and the contrast between grey and white matter is reduced. The motion-corrected images show little blurring and an increased contrast between grey and white matter. The profiles in Figures 5 and 7, taken in the coronal plane across the basal ganglia, show a twofold increase in contrast between white matter and the basal ganglia. Figure 8 shows a slice of Patient A where uptake in the *superior colliculi* is clearly visible in the motion corrected PET images, but not in the non-motion corrected image. Tracer dynamics in the *basal ganglia* of Patient A shows a drop in activity over time in the non-motion corrected images, while the motion corrected images show stable activity.

## Discussion

### PET Image Quality

Both phantom and patient studies show that an MR-based motion correction in simultaneous MR-PET neuroimaging is feasible and consistently delivers improved image quality. A better delineation of small and cortical structures is present in the motion corrected *in vivo* PET images. In particular at the basal ganglia, an increase in contrast compared to the surrounding white matter is observed relative to the non-motion corrected images (Figures 5 and 7). Furthermore, the hippocampus becomes visible in the motion corrected images (Figure 5). Even tiny structures such as the *superior colliculi* become visible (see Figure 8A–C). Comparison of the motion corrected images to the reference images shows that the majority of resolution loss due to motion can be recovered.

The MAF and LORMC method provide comparable results in terms of image quality.

This is related to the fact that the same corrections in combination with the OPA algorithm as well as the same framing are applied for both methods. Surprisingly, this is true also for short frame lengths of single positions close to the sampling rate of the EPI sequence, as found in the analysis of MSD values (Figure 6). This result applies only to reconstructions utilising the OPA algorithm, as it appropriately considers the non-negativity constraint. For the MSD analysis only phantom data were used, as here complete control over intra-frame motion was achieved.

The proposed calculation of normalisation factors, Eq. (8), realised as post-normalisation is of relevance to achieve unbiased, artefact-free images for the LORMC method [26], [31]. Generally, normalisation *after* the process of data binning [31] has significant merit in terms of reduced image noise compared to pre-normalisation. Besides this reduction of noise, the applied post-normalisation procedure overcomes several drawbacks reported elsewhere [8], [17], while the computational burden remains moderate. For example, “*Out-of-FOV Correction*” and “*LOR Discretisation Correction*” as reported in [8], [11] can be completely omitted. The former is appropriately considered due to the statistical weight for each frame λ_ε_ as introduced in Eq. (7). The latter correction is no longer required since our post-normalisation procedure provides an adequately matching normalisation pattern. Also, complications in the calculation of normalisation factors in case of compressed sinogram data using span and mashing inherently disappear for the proposed post-normalisation procedure according to Eq. (8) in combination with OPA [17].

Corrections for Compton scattering in LOR space by applying the Single Scatter Simulation [33] are currently in preparation. The estimated scatter background could simply be combined with the VR as overall additive contribution which can be alternatively considered in the OPA.

### Motion Tracking and Framing

Our results confirm that the errors in the EPI motion estimates consistently reduce with increasing GRAPPA factor. One explanation for this is the reduction of image distortions due to the shortening of the EPI echo train. EPI suffers from severe image distortions, which are a result of phase errors caused by inhomogeneities of the static magnetic field. A portion of these inhomogeneities are caused by the magnetic susceptibility distribution of the head. These change, when head motion occurs, resulting in varying geometric distortions in the EPI images of a time series, reducing the accuracy of the realignment procedure. The long echo train employed in the EPI readout gives ample time for phase errors to accumulate. When the echo train is shortened by using GRAPPA, the distortions in the images are reduced. Using parallel imaging comes at the expense of a lowered signal-to-noise ratio in the EPI data. Whether this penalty is acceptable will depend on the specific design of the fMRI study and the analysis method used. It is nevertheless noted that parallel imaging is widely used in fMRI.

Considering the results of the phantom study, one limitation of the method is likely to be imaging of patients with metal implants. The resulting field inhomogeneities caused by the implants can degrade the accuracy of the motion tracking.

The developed framing algorithm minimises the computational effort which has to be spent on motion correction. In the work of Catana *et al.* [12], motion updates are performed rapidly in the order of a few seconds with subsequent framing of the addressed data at every update. In contrast, our method follows a more efficient approach by utilising framing [11], [13], [14]. Rapid updates may not always be necessary if the patient position is unchanged during parts of the imaging process. Therefore, the acquired data can be usually segmented into larger frames during periods of negligible motion. This reduces the computational burden both in MAF and LORMC reconstruction. In the MAF method the number of frames is reduced, while in the LORMC method it reduces the number of full LOR mapping functions Ψ(*i,j,*ε) which have to be calculated for a minimised number of subject positions. This time is instead invested into taking advantage of the optimal image quality of PRESTO. For example, in typical dynamic brain studies the acquired list mode data is divided into 10–20 subsequent subframes which are reconstructed independently. Each subframe requires about 60 iterations and thus nearly 1 hour calculation time. Therefore, a full dynamic reconstruction is available after 5–10 hours where the reduction factor of two in speed is gained by parallel reconstruction of two subframes simultaneously on the cluster. Generally, the calculation time to provide LORMC data (approximately 1 minute per position) is almost negligible compared to the overall reconstruction time. In contrast, using MAF this scenario gives significantly longer reconstruction times since the motion-triggered subframing usually requires more frames to be reconstructed.

Acquiring motion information through image realignment as performed here with EPI is only possible if multiple MR image volumes are acquired consecutively. In MRI sequences such as for example MP-RAGE, where only a single image volume is acquired in 5–10 minutes, motion information cannot be acquired by image realignment. Another limitation is the frequency of motion detection with an update every 2.2 s. This does not allow one to correct for very rapid head motions such as tremors. However, both limitations can be remedied by including navigators [34], [35] into the sequences. In an MP-RAGE acquisition the motion parameters can be included in each inversion interval to deliver motion information. In an EPI sequence the navigators could be introduced after each slice acquisition, which would allow motion updates in less than 100 ms, albeit at the cost of an increased acquisition time.

EPI image realignment has proven itself to be a reliable motion correction method for functional MRI studies where it is performed routinely as a pre-processing step. Our phantom and *in vivo* studies indicate it is also a suitable method for simultaneous MR-PET motion correction. Especially in the confined space of the BrainPET system, the ability to perform motion correction without any additional hardware offers an advantage. However, in PET motion tracking utilising external tracking systems is widely used [8], [14], [15], [36] and can still be considered the gold standard. Thus, a systematic comparison between motion correction using MR-based tracking and external tracking is of interest and subject to future investigation.

Finally, it should be noted that the effort for the calibration procedure between MR and PET is reduced in the latest generation of whole-body hybrid MR-PET scanners due to their higher integration, and vendor provided calibration procedures.

### Conclusions

The presented method advantageously combines an EPI protocol which delivers highly accurate motion parameters, a new framing approach which minimises data processing time, and a new list mode based fully-3D reconstruction using the PRESTO framework [18] to obtain optimal image quality in moving patients.

With the approach presented here a tool becomes available which allows for motion correction, especially during long PET/MR measurements such as combined neuroreceptor metabolism studies and functional MRI studies examining the multidimensional brain response to pharmaco-challenges or mental stimuli. Also in the case of shorter studies performed in difficult patient populations such as Alzheimer and Tourette patients our method will help to ensure sufficient image quality.

## Supporting Information

Figure S1 **Patient C.** (A) Non-motion corrected, (B) MAF corrected, and (C) LORMC corrected PET images, (D) reference PET image without motion, along with (E) the corresponding MP-RAGE image of Patient B. The profiles (F) along the white lines are shown in the top right corner, patient motion parameters (G) are shown in the bottom right corner.
(TIF)
