# Supplementary figures and images for: MR-Based PET Motion Correction Procedure for Simultaneous MR-PET Neuroimaging of Human Brain
Source: PLoS One. 2012 Nov 12;7(11):e48149. doi: 10.1371/journal.pone.0048149 (PMC3495949; doi:10.1371/journal.pone.0048149)

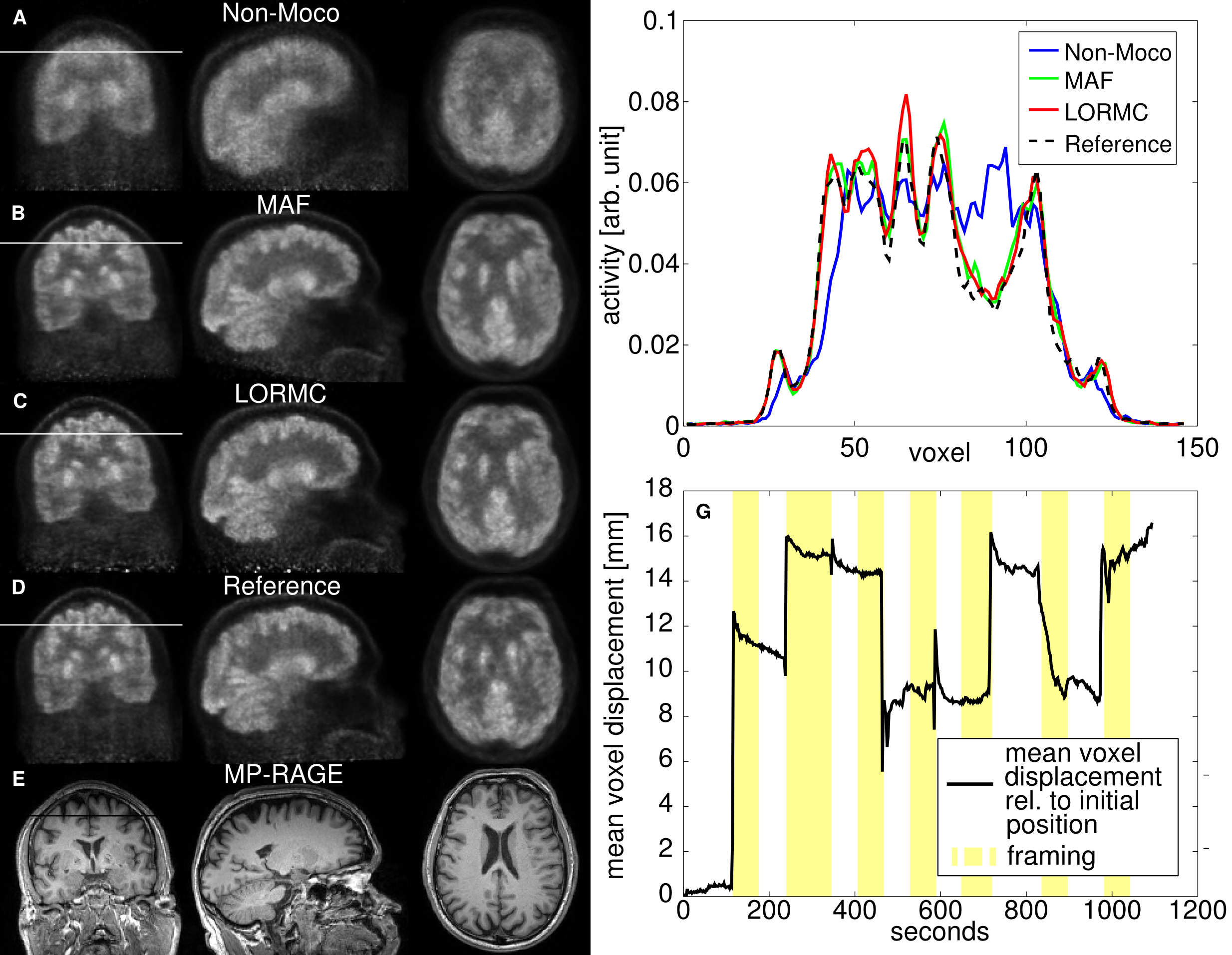

Supplement: Figure S1 — Patient C. (A) Non-motion corrected, (B) MAF corrected, and (C) LORMC corrected PET images, (D) reference PET image without motion, along with (E) the corresponding MP-RAGE image of Patient B. The profiles (F) along the white lines are shown in the top right corner, patient motion parameters (G) are shown in the bottom right corner. (TIF) [file pone.0048149.s001.tif]
